# Oxaliplatin–Biomimetic Magnetic Nanoparticle Assemblies for Colon Cancer-Targeted Chemotherapy: An In Vitro Study

**DOI:** 10.3390/pharmaceutics11080395

**Published:** 2019-08-06

**Authors:** Ylenia Jabalera, Beatriz Garcia-Pinel, Raul Ortiz, Guillermo Iglesias, Laura Cabeza, José Prados, Concepcion Jimenez-Lopez, Consolación Melguizo

**Affiliations:** 1 Department of Microbiology, Sciences School, University of Granada, Campus de Fuentenueva, 18002 Granada, Spain; 2 Institute of Biopathology and Regenerative Medicine (IBIMER), Center of Biomedical Research (CIBM), University of Granada, 18100 Granada, Spain; 3 Department of Anatomy and Embriology, Faculty of Medicine, University of Granada, 18071 Granada, Spain; 4 Instituto de Investigación Biosanitaria IBS.GRANADA, 18012 Granada, Spain

**Keywords:** colon carcinoma, magnetite nanoparticles, MamC, magnetotactic bacteria, nanocarriers, oxaliplatin

## Abstract

Conventional chemotherapy against colorectal cancer (CRC), the third most common cancer in the world, includes oxaliplatin (Oxa) which induces serious unwanted side effects that limit the efficiency of treatment. Therefore, alternative therapeutic approaches are urgently required. In this work, biomimetic magnetic nanoparticles (BMNPs) mediated by MamC were coupled to Oxa to evaluate the potential of the Oxa–BMNP nanoassembly for directed local delivery of the drug as a proof of concept for the future development of targeted chemotherapy against CRC. Electrostatic interactions between Oxa and BMNPs trigger the formation of the nanoassembly and keep it stable at physiological pH. When the BMNPs become neutral at acidic pH values, the Oxa is released, and such a release is greatly potentiated by hyperthermia. The coupling of the drug with the BMNPs improves its toxicity to even higher levels than the soluble drug, probably because of the fast internalization of the nanoassembly by tumor cells through endocytosis. In addition, the BMNPs are cytocompatible and non-hemolytic, providing positive feedback as a proof of concept for the nanoassembly. Our study clearly demonstrates the applicability of Oxa–BMNP in colon cancer and offers a promising nanoassembly for targeted chemotherapy against this type of tumor.

## 1. Introduction

Colorectal cancer (CRC) is the third most common cancer in the world with an estimated 1,096,601 new cases and 551,269 deaths in 2018, respectively [1]. CRC usually develops from focal changes in the colon epithelial tissue (benign, precancerous polyps) to a cancerous polyp, with genetic alterations and their accumulation as one of the most important factors in the development of cancer [2,3]. Other important factors are age, being adolescents and eldest the most susceptible groups (40% of patients are older than 75 years old), gender (more frequent in men), and, in general, diet and lifestyle [2,4,5,6]. Surgical resection of the primary tumor is the main treatment for CRC, followed by adjuvant chemotherapy. The most used chemotherapeutic drugs are 5-fluorouracil (5-FU), oxaliplatin (Oxa), and irinotecan. These drugs are commonly used in combination, mainly due to their synergistic effects and better prognosis, where the most common combinations are FOLFOX (5-FU/leucovorin + Oxa), FOLFIRI (5-FU/leucovorin + irinotecan), and FOLFIR-INOX (5-FU/leucovorin + Oxa + irinotecan) [7,8]. In particular, Oxa is a member of a class of platinum-based compounds that act as alkylating cytotoxic agents. It inhibits DNA replication and it is currently used for the treatment of CRC, also providing antitumor activity in the treatments of pancreatic, gastric, ovarian, bladder, breast, small- and non-small-cell lung, and head and neck cancer [9,10,11]. However, conventional chemotherapy involves delivering drugs with off-target and unwanted side effects. In the case of Oxa, these side effects are progressive peripheral sensory neuropathy, acute mild neuropathy, diarrhea, vomiting, and hematological suppression [12,13]. In addition, factors such as the emergence of resistance (whether intrinsic or acquired), the drug pharmacokinetics, and drug degradation before reaching the tumor tissue limit the efficiency of these treatments [2,14]. In this context, an interesting fact is that nanoparticle (NP)-encapsulated drugs have different pharmacokinetic profiles, dictated by the physical and chemical properties of the NP, compared to those of the free drug [14]. Therefore, targeted chemotherapy is being explored for CRC at the present with the goal of minimizing (or avoiding) secondary effects linked to the systemic distribution of the drug, to increase the efficiency of the treatment and to reduce the cost [15].

In this context, much effort was dedicated to obtaining a good nanocarrier which is biocompatible, which can be directed to the target site, which serves as a multifunctional platform to carry drugs, and which can be released at the tumor site driven by external stimuli. Magnetic nanocarriers, especially magnetite (Fe_3_O_4_), were extensively explored in this regard, as they present many of the advantages listed above plus the fact that, exhibiting low to no toxicity in humans [16,17], they can be directed to the target site through the application of an external magnetic field [16,17,18,19,20]. The efficiency of such magnetic guidance depends on the magnetic moment per particle of the nanocarrier, in which the size of the superparamagnetic, crystalline stoichiometric magnetite nanoparticle (NP) plays a major role [21]. Most of the superparamagnetic NPs (SPIONs) already commercialized are usually smaller than <30 nm; thus, their magnetic moment per particle could be increased if larger NPs were used. Moreover, most of these NPs need to be coated following different post-production procedures with the goal of providing surface functional groups that allow functionalization/release of the relevant molecule based on external stimuli [22]. Such a coating may shelter the magnetic core, thereby also decreasing the efficiency of the magnetic guidance. Moreover, when hyperthermia is also going to be used in the treatment, size becomes important to increase the heating power generated per particle unit mass upon the application of an alternating external magnetic field [23]. In fact, such heating power is generated directly related to the amount of iron in the sample, and it should be as high as possible while keeping both the magnetic field within the acceptable clinical range and a low dose of NPs [23].

While the production of synthetic magnetic NPs of relatively large sizes (>30 nm) could be challenging, magnetosomes produced by magnetotactic bacteria offer a greener alternative [21]. They are remarkable in the context of magnetic properties, since (1) they are larger than most inorganic magnetites produced at room temperature, displaying the largest magnetic moment per particle [21,24], and (2) they behave as excellent hyperthermia agents, thus allowing a reduction of the dose of nanoparticles needed to heat up a tissue [23]. The main drawback in terms of their potential use in clinics is the difficulty of scaling up their production [21]. An alternative to obtain good yields of magnetosome-like nanoparticles without the need of scaling up magnetosome production is the inorganic synthesis of biomimetic magnetic nanoparticles (BMNPs) mediated by magnetosome membrane-associated proteins (MAPs). In this context, magnetite nanoparticles mediated by Mms6 [25,26,27] and MamC [28,29,30] are the ones more thoughtfully studied. These proteins are able to control the kinetics of magnetite nucleation and/or growth, resulting in crystals that are different than those formed inorganically under identical conditions and, therefore, display novel properties. Compared to most Mms6–BMNPs and inorganic (protein-free) magnetic NPs (MNPs), MamC–BMNPs have the largest size and the highest blocking temperature, all of which point to the largest magnetic moment per particle [28]. Moreover, MamC provides functional groups to the surface of the BMNPs, which allows functionalization based on electrostatic interaction. In fact, previous studies from our group demonstrated that, due to these BMNPs being negatively charged at physiological pH, they could electrostatically bind to any molecule that is positively charged at this pH, such as doxorubicin (DOXO), forming stable nanoassemblies. However, as the environmental pH decreases approaching the isoelectric point (iep) of the BMNPs (iep 4.4), this electrostatic bond weakens and DOXO is released [31]. 

Magnetic nanoparticles designed for the delivery of Oxa were previously reported in a few publications. Dutta and Sahu [32] used MNPs incorporated in pectin as nanocarriers for the delivery of Oxa, and demonstrated a sustained release of the drug and excellent cytotoxicity against pancreatic cancer cells. Similarly, a combined radiotherapy and chemotherapy carrier was designed by Munaweera et al. [33] who demonstrated their toxicity against non-small-cell lung cancer. These authors demonstrated that the application of a constant magnetic field favored the location of nanocarriers loaded with drugs in the tumor environment, and a subsequent increase of drug in the tumor. However, to the best of our knowledge, no attempt was made to combine magnetic hyperthermia with the goal of locally increasing the potential toxic effect of an Oxa–NP nanoassembly at the tumor site. The present paper is the first work to explore the functionalization of MamC-mediated BMNPs (hereon referred to as BMNPs) with Oxa and the potential applications of this magnetic nanoassembly (hereon referred to as Oxa–BMNPs) for directed chemotherapy combined with hyperthermia treatments. Firstly, Oxa–BMNPs were synthetized and characterized, and the kinetics of Oxa adsorption/release was studied under different conditions, including hyperthermia. Lastly, BMNP cytocompatibility, Oxa–BMNP toxicity, and cell proliferation in the presence of the nanoassemblies were studied in vitro, with the goal of providing a proof of concept for the potential application of these nanoassemblies in directed chemotherapy against CRC.

## 2. Materials and Methods 

### 2.1. Expression and Purification of MamC and Synthesis of BMNPs 

MamC was expressed and purified as recombinant protein following the protocol described in Reference [28]. *Escherichia coli* TOP10 (Life Technologies: Invitrogen, Grand Island, NY, USA) was transformed with the plasmid pTrcHis-TOPO (Life Technologies: Invitrogen) used as a vector of the MamC protein-coding gene (Mmc1_2265) coupled to a hexahistidine tag coding sequence at its 5’ terminus. Isopropyl-1-thio-β-d-galactopyranoside (IPTG) was added to cultures of these transformed *E. coli* TOP10 to induce protein expression. Once expressed, the purification of the protein was carried out under denaturing conditions by fast protein liquid chromatography (FPLC, GE Healthcare) by using immobilized metal affinity chromatography (IMAC, GE Healthcare, Chicago, IL, USA). Finally, fractions containing MamC were refolded at 4 °C through dialysis by using 1 L of the buffers A (Tris 50 mM, NaCl 150 mM, urea 6 M, pH 8.5) and B (Tris 50 mM, NaCl 150 mM, pH 8.5) as the starting and endpoints, respectively. Buffer A was diluted stepwise with buffer B every 5 h. Finally, the eluate was dialyzed against buffer B overnight. The purity of the protein was evaluated by SDS-PAGE electrophoresis.

The synthesis of BMNPs was carried out at 25 °C and 1 atm total pressure from oxygen-free solutions (protocol described in Reference [28]) containing 3.5 mM NaHCO_3_, 3.5 mM Na_2_CO_3_, 2.78 mM Fe(ClO_4_)_2_, 5.56 mM FeCl_3_,and 10 μg/mL recombinant MamC, at a pH value of 9. All experiments were done under anoxic conditions inside an anaerobic Coy chamber (96% N_2_/4% H_2_). Samples were incubated for 30 days and then the solids were magnetically concentrated, washed three times with deoxygenated Milli-Q water (50 mL), and stored in HEPES buffer (pH 7.4) inside the Coy Chamber at 25 °C until further analyses.

### 2.2. Nanoparticle Characterization

Powder X-ray diffraction (XRD) analysis was carried by an Xpert Pro X-ray diffractometer (PANalytical; Almelo, The Netherlands) using Cu Kα radiation (scan range 20–60° in 2θ, 0.01°/step; 3 s/step). Identification of the precipitates was performed using the XPowder software [34]. Transmission electron microscopy (TEM) analyses were performed with a STEM Philips Model CM20 microscope on ultrathin sections (50–70 nm) prepared by embedding the nanoparticles in Embed 812 resin and then cutting them using a Reichert Ultracut S microtome (Leica Microsystems GmbH, Wetzlar, Germany). The size of the crystals was measured on more than 1000 nanoparticles using the ImageJ 1.47 software, and size distribution curves were determined from these measurements. Basic mineral characterization is included in the present manuscript, while further characterization of the BMNPs [thermogravimetric analyses, specific surface area by Brunauer–Emmett–Teller (BET), and hysteresis cycle] can be found in [28,31]. The BMNPs used in the present study are superparamagnetic, contain up to 5 wt% MamC, and have an isoelectric point of ~4.4, specific surface area of ~90 m^2^/g, and blocking temperature of ~145 K [28].

### 2.3. Oxa–BMNP Nanoassemblies

Oxaliplatin was purchased from Sigma Aldrich. The kinetics of Oxa adsorption on BMNPs over time was performed by mixing aliquots of 5 mg of BMNPs from the HEPES aqueous suspension with 1 mL of Oxa (1 mg/mL) in HEPES buffer. Mixtures were incubated at 25 °C for different time intervals, up to 96 h, under continuous stirring. Then, the Oxa–BMNP nanoassemblies (Oxa–BMNPs) were magnetically collected and washed twice with 1 mL of HEPES buffer. The amount of non-adsorbed Oxa was indirectly measured by ultraviolet–visible light (UV–Vis) spectroscopy at a wavelength of 240 nm in all the supernatant collected, including those resulting after washing the pellet. The amount of non-adsorbed Oxa (C_e_) and the amount of adsorbed Oxa per mass unit of adsorbent (Q) was calculated from these measurements, following the protocol described in Reference [31]. The molar absorptivity of Oxa in HEPES buffer at 240 nm was determined as 1702.84 L∙mol^−1^ (*R*^2^ = 0.9986) from the slope of a standard calibration straight line. More than three replicas were performed per experiment. The standard deviation of the absorbance measurements was used to calculate the error in the concentration of Oxa in the supernatant ([Oxa]_sn_).

The adsorption isotherms were determined by mixing 5 mg of BMNPs with 1 mL of different concentrations of Oxa, up to 2 mg/mL in HEPES buffer. More than 10 independent experiments, each one replicated at least three times, were performed at different initial concentrations of Oxa. Mixtures were incubated at 25 °C for 72 h (equilibrium time determined from the adsorption kinetics). The data were fit to the models of Langmuir and Langmuir–Freundlich.

Stock suspensions of BMNPs and Oxa-BMNPs were prepared in 15 mL of oxygen-free NaClO_4_ (10 mM). Aliquots of 200 μL from each stock were suspended in flasks containing oxygen-free NaClO_4_ (final volume of 10 mL/flask), and the pH was adjusted to either 5.0 (four flasks) or 7.4 (four flasks). Samples were sonicated for 2 min, and the electrophoretic mobility was immediately measured (nine replicas per sample). The ζ-potential values were calculated using Malvern Zetasizer software (Malvern Instruments, Malvern, Worcestershire, UK).

Zero-field cooling (ZFC) and field cooling (FC) measurements were performed by a superconducting quantum interference device (SQUID) 5 T magnetometer (Quantum Design MPMS XL, USA). Samples were loaded under argon flow and immediately cooled in a zero applied field to 5 K. Then, a 500-Oe magnetic field was applied and samples were heated up to 300 K and then allowed to cool from 300 K without turning the field off. M(T) curves were normalized by the mass (g) analyzed and by their respective magnetization value at 300 K. Blocking temperature (T_B_) was calculated as the maximum magnetization values of the ZFC curves.

Drug release from Oxa–BMNPs was analyzed at pH 7.4 and pH 5.0 at a controlled temperature of 37 ± 1 °C in a thermostatic bath. Also, Oxa–BMNPs at pH 5.0 were exposed to an alternate magnetic field set to raise the temperature of the suspensions to 43 ± 1 °C (under conditions detailed below) to evaluate the potential triggering effect of hyperthermia on the release. As a control experiment, identically prepared samples were kept in a thermostatic bath set at 43 ± 1 °C and the Oxa release was also analyzed. To do all these experiments, the Oxa–BMNPs were suspended in HEPES buffer (10 mM, 1 mL, pH 7.4) containing NaCl 0.15 M, or in citrate buffer (composed by 35% of citric acid monohydrate 0.1 M and 65% trisodium citrate dihydrate 0.1 M, pH 5.0). Suspensions were incubated at 37 °C, 150 rpm for different time intervals up to 96 h (pH 7.4) and up to 3 h (pH 5). Samples were centrifuged at 10,000 rpm for 5 min and supernatants were analyzed by UV–Vis spectroscopy. The release efficiency (D_R_) was calculated as determined by Reference [31]. The hyperthermia tests were carried out using a Roger-type oscillator set at 197 kHz, and a magnetic field strength of 18 kA/m measured at the center of the coil with a NanoScience Laboratories Ltd., Probe (Newcastle, UK), with 10-µT resolution. A seven-turn coil (20 mm in diameter and 45 mm in length) made of a 4-mm water cooled copper tube was connected to the oscillator. A fiber optic thermometer (Optocon AG, Dresden, Germany) was placed into a 1.5-mL Eppendorf tube containing 1 mL of 5 mg/mL Oxa–BMNPs. The sample was previously pre-thermostated at 37 ± 1 °C and thermally insulated. Then, the abovementioned conditions of frequency and magnetic field strength were set to reach a hyperthermia temperature of 43 °C during all the release tests [35].

### 2.4. Cell Culturing

The human cell lines CCD-18 (colon fibroblast), T-84 (colon carcinoma), HT-29 (colon adenocarcinoma), SW480 (colon adenocarcinoma Duke’s type B), and HCT-15 (colon adenocarcinoma Duke’s type C), and the murine cell lines RAW 264.7 (macrophages) and MC-38 (colon adenocarcinoma) were obtained from the American Type Culture Collection (ATCC), and the Technical Services of Granada University, kindly provided by Dr. J. Scholl (Public Health Service, National Institutes of Health, Bethesda, MD, USA), respectively. All cell lines were grown in Dulbecco’s modified Eagle’s medium (DMEM), supplemented with 10% fetal bovine serum (FBS) and 1% antibiotics (penicillin–streptomycin) (Sigma-Aldrich, Madrid, Spain). Roswell Park Memorial Institute (RPMI)-1640 medium supplemented with 10% FBS and 1% antibiotics (penicillin–streptomycin) (Sigma-Aldrich, Madrid, Spain) was used for the white blood cell culture. Cells were maintained in a monolayer culture at 37°C in an atmosphere containing 5% CO_2_. 

### 2.5. In Vitro Proliferation Assays 

Cells were seeded in 48-well plates (5 × 10^3^ cells/well for T-84, CCD-18, and HCT-15, 1.5 × 10^4^cells/wellfor HT-29, 4 × 10^3^ cells/well for SW480, and 3 × 10^3^ cells/well for MC-38,) and incubated overnight. Then, the different treatments, BMNPs (as control), FeCl_3_, Oxa–BMNPs, and soluble Oxa, were administered in a growing drug dose range. After 72 h, the viability was determined by a sulforhodamine B (SRB) protocol described previously [36]. Briefly, the cells were fixed with cold trichloroacetic acid for 20 min at 4°C and washed three times with distilled water. After drying, the cells were stained with SBR (Sigma Aldrich, Madrid, Spain) for 20 min at room temperature while stirring, washed three times with acetic acid 1%, and then the dye was resuspended in Trizma^®^ (Sigma-Aldrich, Madrid, Spain). Finally, the dye was measured at 492 nm in a Titertek Multiscan Colorimeter (Flow, Irvine, CA, USA), and the relative proliferation percentage with respect to control was obtained.

### 2.6. Blood Cell Compatibility of MamC-Mediated Magnetite Nanoparticles

#### 2.6.1. Red Blood Cell Assay

This test was performed to observe the potential lytic effect of BMNPs on red blood cells. A modified protocol was used [37]. Ethylenediaminetetraacetic acid (EDTA) tubes were used to collect blood from a healthy human donor. After centrifugation (1600 rpm, 5 min) the plasma was discarded, and erythrocytes were washed and centrifuged twice in 150 mM NaCl. The pellet obtained was resuspended in phosphate buffered saline (PBS, pH 7.4). After that, the erythrocytes were diluted by adding PBS (1:50). A volume of 190 µL of this suspension was placed in each well of a V-bottom 96-well plate. A volume of 10 µL of BMNPs was added from stocks of different initial concentrations, to allow a final BMNP concentration in the wells ranging from 5 to 500 µg/mL. Aliquots of 10 µL of 20% Triton X-100 and PBS pH 7.4 were used for positive and negative controls, respectively. Then, the samples were maintained for 1 h at 37 °C under stirring (15 rpm) and, finally, the plate was centrifuged. A volume of 100 µL of the supernatant from each well was recovered, placed in a new flat-bottomed 96-well plate, and measured at 492 nm. The percentage of hemolysis (%HR) was calculated with the following formula:

$$\%HR=\frac{Abs\left(sample\right)-Abs\left(-control\right)}{Abs\left(+control\right)-Abs\left(-control\right)}\times 100.$$

After treatment, samples were observed with a light microscope and photographed (Leica DM IL LED).

#### 2.6.2. White Blood Cell Proliferation Assay

EDTA tubes were used to collect human blood from a healthy donor. In addition, Histopaque^®^-1077 (Sigma–Aldrich, Madrid, Spain) was added at the same volume and then the tubes were centrifuged (1400 rpm, 30 min). Slowly, the upper layer of blood plasma was removed, and the white blood cells (WBC) (buffy coat) of the intermediate layer were collected. Next, in a new 15-mL tube, the cells were washed by centrifugation (2200 rpm, 10 min) by using 10 mL of PBS. After discarding the supernatant, the cells were resuspended in RPMI-1640 supplemented with 10% FBS and 1% antibiotics (penicillin–streptomycin) (Sigma-Aldrich, Madrid, Spain) [38]. Cells were seeded (2 × 10^4^ cells/well) as described previously [39], and 10 µL of BMNPs were added to each well from stock solutions of different concentrations to reach a final concentration of 5 to 500 µg/mL BMNPs. Then, the experiments were incubated for 1 and 12 h at 37 °C and 5% CO_2_ in a humidified atmosphere. After that, a Cell Counting Kit-8 (CCK-8) (Dojindo Laboratories, Kumamoto, Japan) was added to reach a 10% final concentration to measure the cell viability. The samples were incubated for 4 h and, finally, the sample absorbance was measured at 450 nm.

#### 2.6.3. Cell Cytotoxicity of MamC-Mediated Magnetite NPs in RAW 264.7 Cells

The RAW 264.7 cell line was used to test the toxicity of BMNPs in macrophages. A density of 7500 cells/well was seeded in a 96-well plate in a volume of 90 µL and treated with BMNPs (in 10 µL) to a range of different concentrations for 1 and 12 h. Then, cell viability was determined by adding CCK-8 reagent to each well to reach a final concentration of 10%. After 4 h of incubation, absorbance was measured at 450 nm (Titertek Multiscan Colorimeter; Flow, Irvine, CA, USA).

### 2.7. Internalization and Functionality Tests of BMNPs

#### 2.7.1. Cell Staining for Iron Determination

To observe the presence of BMNPs in tumor cells, T-84 and RAW 264.7 macrophages cells were seeded on eight-well chamber slides and allowed to attach. Then, BMNPs and FeCl_3_ were added to the cell cultures from 10 to 100 µg/μL final iron concentration. After 24 and 48 h, the cells were visualized by Prussian blue staining for iron detection following a previously described protocol [39]. The samples were observed with a light microscope (Leica DM IL LED).

#### 2.7.2. Transmission Electron Microscopy Assays

Transmission electron microscopy (TEM) was performed to confirm the internalization of BMNPs into tumor cells. For that, T-84 cells were treated with BMNPs for 15 min and 24 h at a final iron concentration in culture media of 10 and 100 µg/μL. Then, cells were collected and centrifuged, and the pellet was immediately fixed and further processed as described previously [39].

#### 2.7.3. Cell Migration Assay

The migration of cells treated with BMNPs under a magnetic field was determined. Cell lines were seeded in six-well plates at a density of 3 × 10^5^ cells/well and exposed to 10 and 100 µg/mL BMNPs and FeCl_3_ at the equivalent iron concentration for 24 h. Once the treatment time (6 h) elapsed, the medium was removed and the cells were detached and reseeded in Petri dishes with 2 mL of fresh medium. All treatments were performed in duplicate, and a magnet was placed under one of the plates of each treatment. After 24 h, cells were fixed and stained as described previously in Section 2.5 to observe the distribution of the cells in the dishes. Untreated cells were used as a negative control.

### 2.8. Statistical Analysis 

Statistical analysis was performed by using the Student’s *t*-test with the Statistical Package for the Social Sciences (SPSS) v.20. All the results are presented as means ± standard deviation (SD). Data with *p* < 0.05 were considered as statistically significant.

## 3. Results

### 3.1. BMNPs and Oxa–BMNP Nanoassemblies 

**Figure 1:**
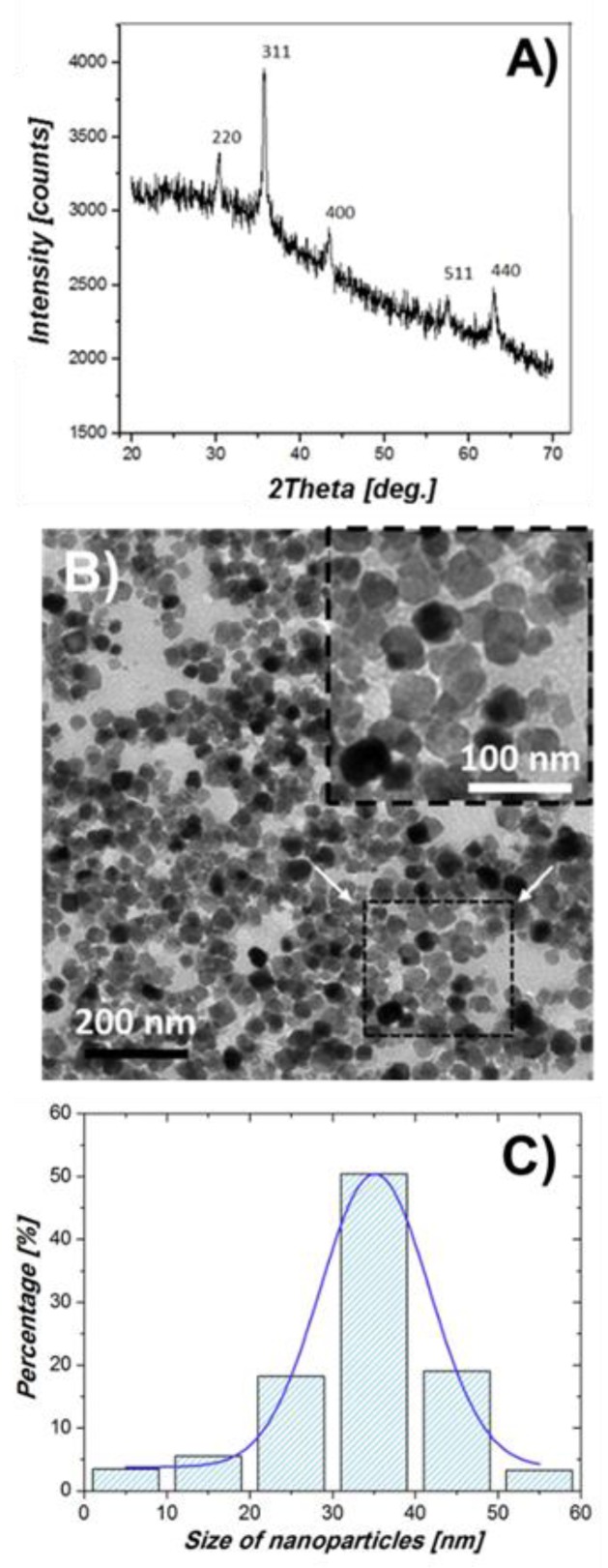
Magnetite crystals synthesized in the presence of MamC (10 μg/mL) (biomimetic magnetic nanoparticles, BMNPs): (**A**) X-ray diffraction (XRD); (**B**) transmission electron microscopy (TEM) image and magnification of a selected area; (**C**) crystal size distribution.

XRD analyses revealed that the solid comprised 100% magnetite, with the main reflection for magnetite being the 311 (d-spacing = 2.530 Å, for Cu Kα radiation, 2θ = 35.44°). TEM micrographs show that most BMNPs were isometric crystals with well-developed faces and a size ranging from 20 to 50 nm, with an average crystal size of 34 ± 10 nm (Figure 1). 

**Figure 2:**
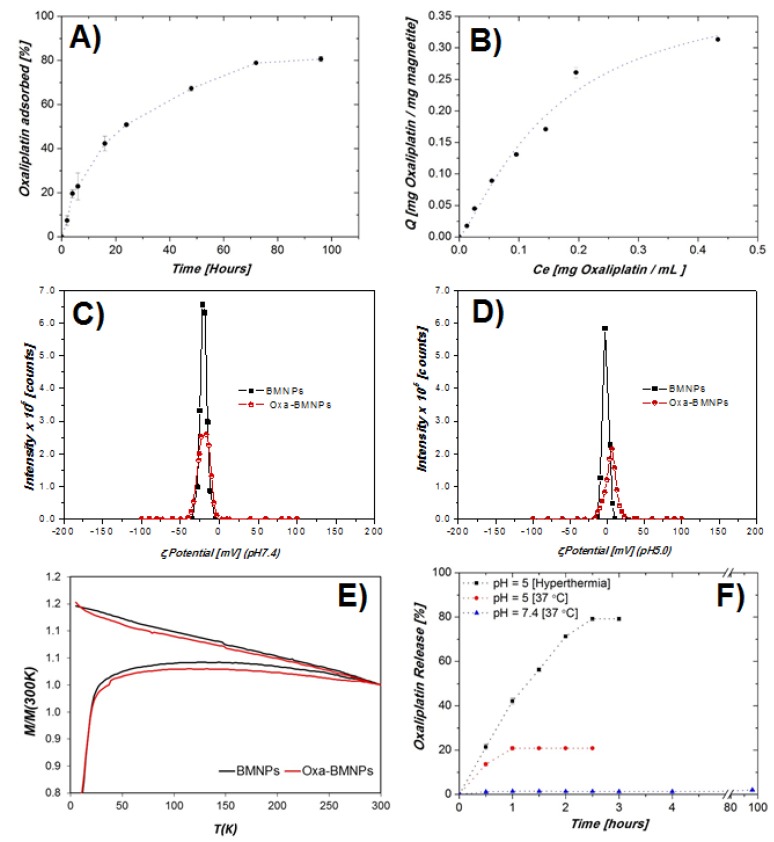
(**A**) Adsorption kinetics and (**B**) adsorption isotherm of oxaliplatin (Oxa) on BMNPs. The line represents the non-linear weighted least-squares (NWLS) fitting of the experimental data according to the Langmuir–Freundlich (LF) model. The vertical error bars are smaller than the symbol. ζ potential calculated for both BMNPs and Oxa-BMNPs at pH values of (**C**) 7.4 and (**D**) 5.0. (**E**) Zero-field cooling (ZFC) and field cooling (FC) measurements for BMNPs and Oxa-BMNPs. (**F**) Kinetics of Oxa release from loaded Oxa–BMNPs over time at physiological pH (pH 7.4) and at acidic pH (pH 5) at 25 °C, also under hyperthermia conditions.

The kinetics of Oxa adsorption over time shows that the system reached equilibrium (no net adsorption/desorption) at about 72 h (Figure 2A). The amount of adsorbed drug per amount of BMNPs (*Q*) increased with the equilibrium concentration of Oxa in the supernatant (C_e_) at a higher rate at the lowest C_e_ values. Such a rate decreased as C_e_ increased (Figure 2B). The adsorption isotherm only adjusted to the Langmuir–Freundlich (LF) models (*R*^2^ = 0.97579), showing a drug loading capacity (*Q_max_*) of 0.41 ± 0.08 mg Oxa/mg magnetite. The effects of energetic heterogeneity of the nanoparticle surface and the cooperativity between Oxa molecules were accounted in this LF model. The values of the LF affinity constant (*K_LF_*) and cooperativity coefficient (*r*) parameters, calculated by means of this model, were of 6 ± 2 mL/mg magnetite and 1.3 ± 0.3, respectively.

The ζ potential values for both BMNPs and Oxa–BMNPs indicate that both particles were negatively charged at physiological pH (ζ potential of BMNPs = −20 ± 3 mV; ζ potential of Oxa–BMNPs = −19.7 ± 0.5 mV; Figure 2C), contributing to the reciprocal repulsion that, in turn, promoted the stability of the suspension. At a pH value of 5.0, only BMNPs remained slightly negatively charged (ζ potential = −2.4 ± 0.6 mV), while Oxa–BMNPs became positively charged (ζ potential = 4.3 ± 0.7 mV; Figure 2D). Such a positive surface charge of the nanoassembly would favor its interaction with the negatively charged cell membranes.

The coupling of Oxa to BMNPs to form Oxa–BMNP nanoassemblies slightly reduced the magnetization of the nanoassembly (Figure 2E), probably due to the non-magnetic coating. Nevertheless, there were no significative differences in the T_B_ values of both BMNPs (126 K) and Oxa–BMNPs (130 K), which were in any case higher than that of inorganic magnetic nanoparticles produced under identical conditions [28], which is indicative of a larger magnetic moment per particle of the former.

The release of Oxa at physiological pH (pH 7.4) was practically negligible, with release efficiency (*D_R_*) values at 24 h that did not exceed the 2% of the initially adsorbed Oxa (Figure 2C). At acidic pH, ~20% of the initially adsorbed Oxa was released from the BMNPs in 1 h. Longer times did not increase D_R_ (~21%). However, when hyperthermia was in play, D_R_ values were always higher compared to those obtained without hyperthermia and increased with time up to 2.5 h, where D_R_ reached a value of ~80%. This last result shows a triggering effect on the drug release at acidic pH values enhanced by hyperthermia, thus demonstrating the existing synergy between the weakening of the electrostatic forces that kept the Oxa bound to the BMNPs and the hyperthermia. This is important, since this would make it possible to reduce both the dose of the drug and the frequency of drug administration.

### 3.2. In Vitro Proliferation Assays

**Figure 3:**
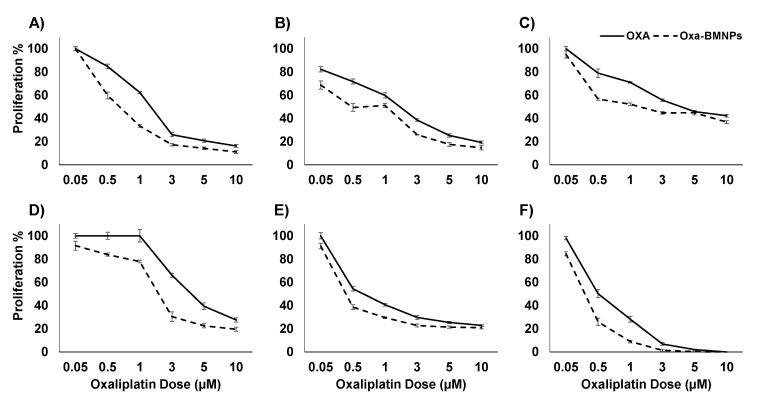
Cell proliferation assay of colon (tumor and non-tumor) cell lines treated with soluble Oxa and Oxa–BMNP. The percentages of proliferation of HT29 (**A**), SW480 (**B**), HCT15 (**C**), MC38 (**D**), T84 (**E**), and CCD18 (**F**) cell lines obtained using the sulforhodamine B (SRB) assay are shown. Data represent the mean values ± SD of triplicate cultures.

Treatment with soluble Oxa and Oxa–BMNPs in colon cancer cells showed significant cytotoxicity in all analyzed colon cancer cell lines (Figure 3). Interestingly, Oxa–BMNPs induced a greater reduction in IC_50_ compared to that of free Oxa (*p* < 0.05). In fact, Oxa–BMNPs induced a more than two fold decrease in IC_50_ of HT-29, SW480, and T-84 cells, reaching values of 2.79, 1.92, and 0.67 μM, respectively. Furthermore, the use of Oxa–BMNPs allowed a significant decrease in IC_50_ even in the HCT-15 resistant cell line (from 1.91 to 1.03 μM). However, a lower reduction in IC_50_ was observed in CCD18 human colon fibroblast cells (Table 1).

**Figure 4:**
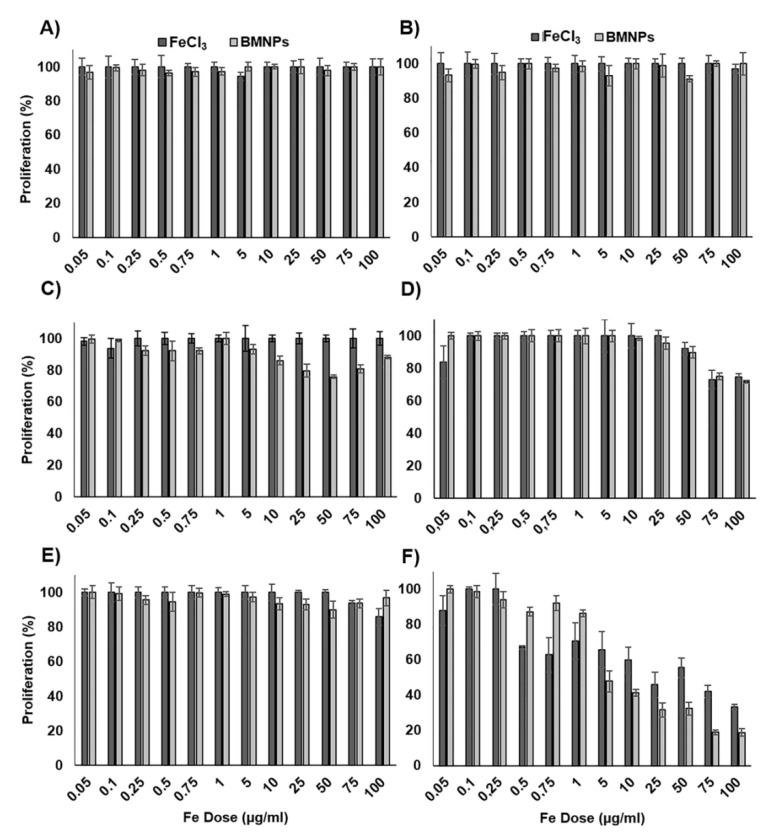
Cell proliferation assay of colon cell lines treated with BMNP and FeCl_3_. The cell lines HT29 (**A**), SW480 (**B**), HCT15 (**C**), MC38 (**D**), T84 (**E**), and CCD18 (**F**) were exposed to increasing concentrations of Fe from 0.05 to 100 µg/mL from FeCl_3_ and BMNPs for 72 h. The graphs represent the percentages of proliferation of all the cell lines obtained using the SRB assay. Data represent the mean values ± SD of triplicate cultures.

On the other hand, FeCl_3_ and BMNPs (without Oxa) showed a relative lack of toxicity in all cell lines, suggesting a high degree of cytocompatibility. In fact, HT-29 and SW480 cell lines did not show a significant reduction in cell viability (*p* > 0.05) compared to controls at all doses of FeCl_3_ and BMNPs tested (Figure 4). Only at the highest doses of FeCl_3_ and BMNPs was there a low decrease in cell viability in HCT-15 and MC38 cell lines. On the other hand, CCD-18 cells were more sensitive to FeCl_3_ and BMNPs, showing a decrease in cell viability at doses greater than 0.5 μg/mL.

### 3.3. BMNP Internalization

**Figure 5:**
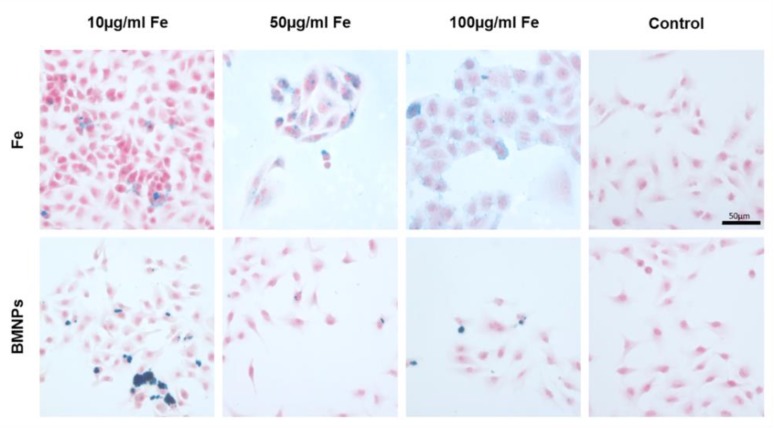
Internalization of BMNPs in colon cancer cells. Representative images of T-84 human cancer cells exposed to different concentrations of BMNPs and Fe and stained with Prussian blue.

**Figure 6:**
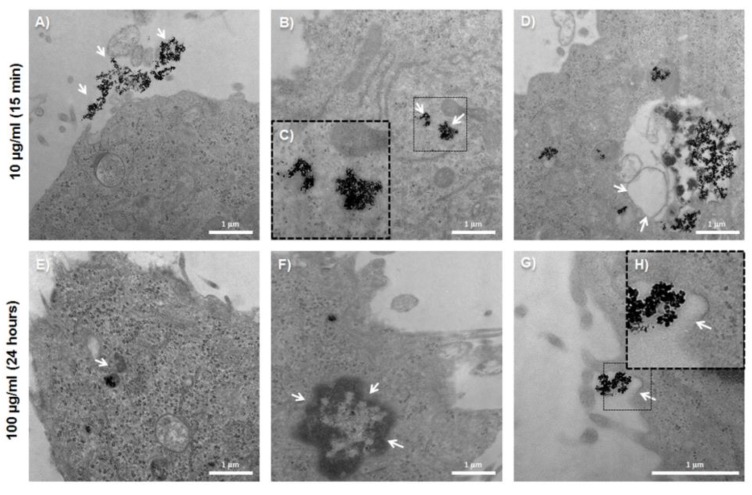
Transmission electron microscopy analysis of the BMNP internalization in colon cancer cells. Representative images the T-84 colon tumor cells showing extracellular (**A**) and intracellular (**B**) magnetic NPs (arrows). Magnification of the image (**C**) in which an outline around the intracellular BMNPs is shown, suggesting that it is inside a vesicle. Multivesicular body (**D**). Intracellular NPs surrounded by a membrane (**E**). Deteriorated cell nucleus (**F**). The dark border that is observed could be a sign of clathrin-mediated endocytosis (**G** and **H**).

To evaluate the ability of the T-84 colon tumor cell line internalizing the BMNPs, Prussian blue staining was performed at different concentrations of BMNPs and FeCl_3_, obtaining a positive staining in all cases (Figure 5). A dose-dependent staining was observed. To confirm the internalization of the BMNPs and their intracellular location, TEM images were obtained (Figure 6), from which BMNPs were observed inside the cell from the first 15 min of exposition time at the lower dose of 10 μg/mL. All BMNPs were observed in the cytoplasm, either free or apparently surrounded by a membrane, while they were not observed in any other subcellular organelles. In Figure 6C, an outline around the BMNPs is shown suggesting that they are surrounded by a membrane. This is more evident at higher concentrations (100 μg/mL) (Figure 6E). A dark border on the endocytosing membrane may be observed in Figure 6G, which is usually typical of clathrin-mediated endocytosis. Briefly, the cellular uptake of BMNPs began only 15 min after inoculation and continued to be internalized 24 h later.

### 3.4. Cell Migration under a Magnetic Field In Vitro

**Figure 7:**
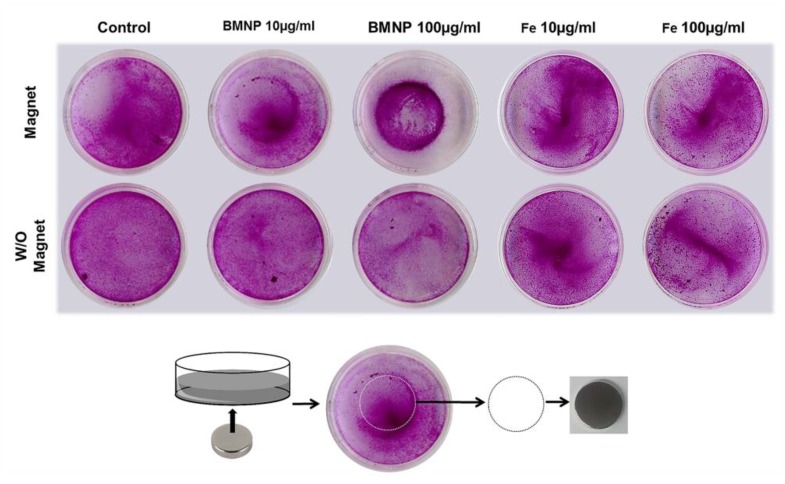
Magnetic migration assay. Representative image of colon cancer cells (HCT15) exposed to different concentrations of BMNPs and Fe and stained with SRB. Migration of the cells after treatments was evaluated in the presence or absence of a magnet. Schematic image represents how the circumference of the magnet (discontinuous line) corresponds with the cell clustering seen in the previous image.

Colon cells were treated with 10 and 100 µg/mL FeCl_3_ and BMNPs, detached, and reseeded with or without the presence of a magnet with round shape. The suspension of cells treated with BMNPs migrated in a dose-dependent manner around the periphery of the magnet, obtaining a circular pattern on the surface of the petri dish that we observed through SRB staining (Figure 7 and Supplementary Materials Figure S1), indicating that once the NPs were internalized, they did not lose their magnetic potential. This circular pattern was observed only slightly in the case of FeCl_3_ treatment, even at the highest dose. As it can be seen in the Figure 7, no round halo was produced in the case of the petri dishes that were not exposed to the magnet or in the negative controls with or without magnet, which means that migration was produced exclusively by the presence of iron in the BMNPs. 

### 3.5. BMNP Biocompatibility in Blood Cells

**Figure 8:**
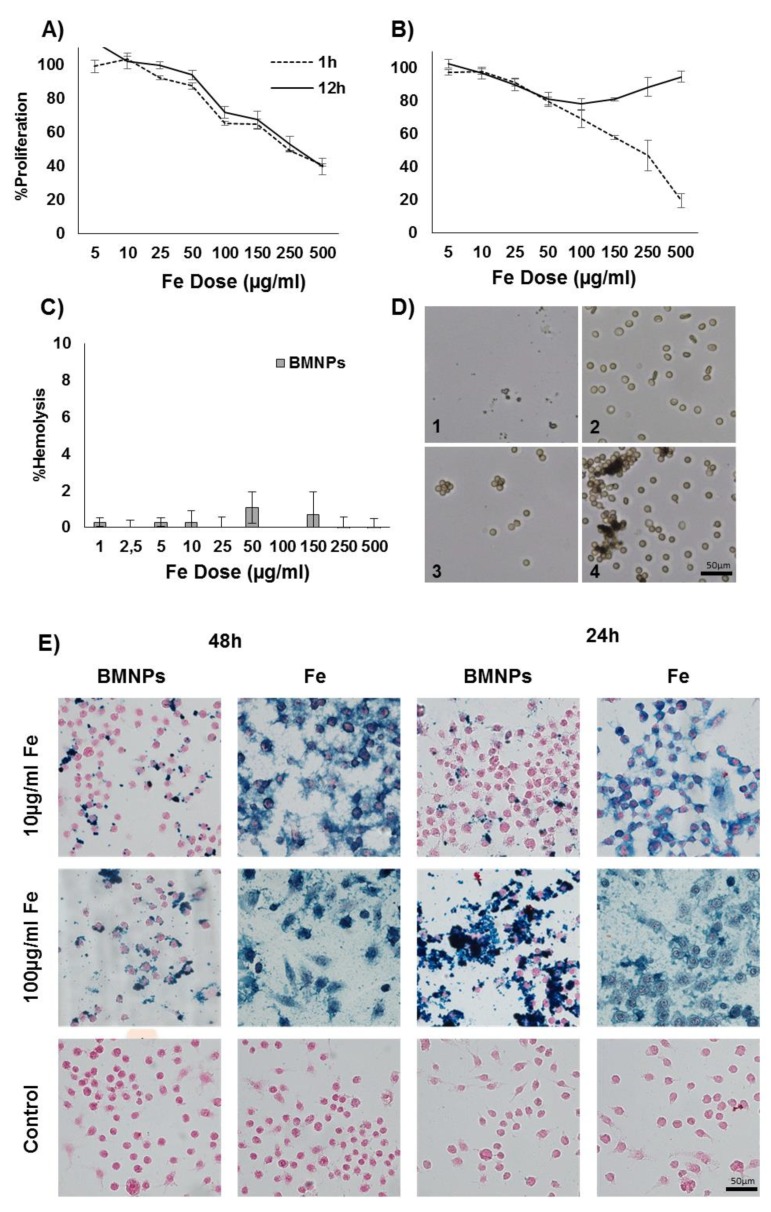
BMNP biocompatibility with blood cells. (**A**) Toxicity test in the cell line of RAW 264.7 macrophages. (**B**) Toxicity test in white blood cells (WBCs). (**C**) Hemolysis test. Data represent the mean values ± SD of triplicate samples. (**D**) Images of optical microscopy of erythrocytes after treatment. 1. Positive control, 2. negative control, 3. 25 μg/mL Fe, and 4. 150 μg/mL Fe. (**E**) Prussian blue staining of RAW 264.7 cell line.

A significant decrease in the viability of macrophages (*p* < 0.05) was observed depending on the BMNP doses administered at both 1 and 12 h (Figure 8A), with no statistically significant differences (*p* > 0.05) in terms of viability among these times. As shown in Figure 8E, a great cellular internalization of BMNPs and also of FeCl_3_ was observed by Prussian blue staining, based on the highly positive, dose-dependent staining of cells treated at BMNP doses of 10 and 100 μg/mL at 24 and 48 h.

In relation to the WBCs, a decrease in cell viability was observed at 1 h of treatment depending on the dose; however, after 12 h of treatment, the cell viability recovered, reaching full recovery at high doses (Figure 8B). Finally, the tests on red blood cells showed that there was no significant percentage of hemolysis in the range of doses tested (percentage of hemolysis <2%) (Figure 8C). Despite this, BMNPs produced a dose-dependent agglutination of the erythrocytes by adhering to these NPs (Figure 8D).

## 4. Discussion

Our results demonstrate the high loading capacity for Oxa that our BMNPs have. Actually, the *Q_max_* value determined from this study (0.41 mg Oxa/mg magnetite) is significantly higher than that determined for Oxa adsorption on other materials, such as hydroxyapatite (0.0491 mg Oxa/g hydroxyapatite [9]). Direct comparisons cannot be done because no data on particle size and/or surface charge were provided in this study. Since no other data of Oxa adsorption on magnetite NPs (or any other NP) are available at the present, comparisons of the *Q_max_* of the present study were done in reference to other studies involving either the BMNPs used here or other molecules in a variety of nanocarriers. The *Q_max_* value determined here is comparable to, although a little lower than, the *Q_max_* determined for the adsorption of doxorubicin (DOXO) on the same BMNPs (0.69 ± 0.03 mg DOXO/mg magnetite [31]) and similar to that for DOXO adsorption on citrate-coated apatite (0.41 [40]), citrate-coated carbonated apatite (0.44 [40]), and superparamagnetic iron-doped apatite nanocrystals (0.45 [40]), as well as that for graphene oxide NPs (1.43 [41]). Such a strong loading capacity could be explained, firstly, by means of electrostatic interactions between Oxa and the magnetite surface, which is negatively charged at a pH of 7.2 [31]. These electrostatic interactions were claimed to be responsible for the formation of DOXO–BMNPs described in Reference [31] and were also responsible for the stability of the Oxa–BMNPs at physiological pH values. Moreover, when these electrostatic interactions weaken as the BMNPs get closer to their isoelectric point (pH 4.4 [31]), the Oxa is released. In fact, Oxa release at physiological pH was practically negligible (*D_R_* < 2%), while this release exceeded 20% at pH 5 (or >80% with hyperthermia), the pH mimicking the conditions in the endosome–lysosome compartment [42] (Figure 2C). This is an important finding for potential clinical application, since no Oxa release is expected in the blood stream until the target tumoral (acidic) environment is reached. Our hypothesis is that, when the pH value of the microenvironment decreases, approximating the iep of the BMNPs (pH 4.4 [31]), the electrostatic interaction between Oxa and the –COO^−^ weakens, and the electrostatic repulsions between adsorbed adjacent Oxa molecules become stronger, facilitating Oxa desorption. However, according to ζ potential values, at pH 5.0, BMNPs are still slightly negatively charged and, therefore, able to keep Oxa bound to their surfaces. This further and faster release of the Oxa molecules that do not desorb is favored by hyperthermia. The rotation of the BMNPs and the temperature increase induced by hyperthermia probably help the shedding of further Oxa molecules. Indeed, it is well known that if the surface to which the molecule is bonded is heated (in this case, as a result of hyperthermia), the energy transferred to the adsorbed species promotes its desorption. Triggering of drug release due to hyperthermia was previously observed for other drugs [43].

However, not only electrostatic interactions between the Oxa and the BMNPs could be argued for such a high *Q_max_*, since the high *r* coefficient (1.3 ± 0.3 mL/mg magnetite) indicates a strong positive cooperativity between the molecules of Oxa during the adsorption process, as a pathway to decrease the adsorption energy. Therefore, the adsorption of an Oxa molecule onto the BMNP surface lowers the energy for the adsorption of the next molecule. Such a cooperativity between molecules in the context of the adsorption was previously described in DOXO adsorption on BMNPs [31] and on citrate-coated apatite nanocrystals [40]. As a result, only the LF model, which accounts for the cooperativity between Oxa molecules and introduces the effects of energetic heterogeneity of the surface, fit the experimental *Q* data (*R*^2^ = 0.97579). In this context, the heterogeneity of the surface is intrinsic to the BMNPs, since distinct magnetite crystal faces with different ionic configurations (and, thus, different net surface charges) are expressed in the final morphology of BMNPs.

Toxicity data from this study evidence, for the first time, the utility of BMNPs to transport, release, and increase the Oxa antitumoral effect in colon cancer cells. Few previous studies analyzed the use and applicability of magnetotactic bacteria or their magnetosomes in colon cancer. For instance, Felfoul et al. [44] analyzed the migration of magnetotactic bacteria using magnetic guides toward the hypoxic regions of HCT116 colorectal xenografts, and the compatibility of magnetosomes using the Colo205 cancer line was also demonstrated [45]. However, our data show that the use of Oxa–BMNP nanoassemblies allowed roughly doubling the drug effect in HT-29, SW480, and T-84 colon cancer cell lines. Moreover, a relevant effect was that observed in resistant colon cancer cell lines such as HCT-15, in which the use of Oxa–BMNPs decreased the Oxa IC_50_ (1.85-fold). This therapeutic advantage could be related to the easy BMNPs internalization by the colon cancer cells assayed. In fact, cellular uptake occurred only after 15 min of exposure, even at the low dose used (10 μg/mL Fe). As shown in TEM analyses, the internalization process resulted in structures surrounded by a membrane. The dark border observed around the BMNPs was widely cited in the literature as typical of clathrin-mediated endocytosis, suggesting that BMNPs could be internalized by this mechanism [46,47].

BMNPs did not show toxicity in cell cultures in most of the doses and cell lines tested, and only for the highest doses (>75 μg/mL) was some toxicity observed in some cell lines. Only CCD-18 cells showed a significant decrease in proliferation at the BMNP dose of 0.5 μg/mL, which could be explained, on one hand, by the formation of large BMNP agglomerations upon contact with the medium [48] and, on the other, by a special sensibility of this cell line to Fe, which was confirmed by previous studies [49], in which some toxicity was induced when cells from this cell line were treated with FeCl_3_. Interestingly, BMNPs were also able to respond to an external magnetic field even once internalized, which represents a therapeutic advantage. Only cells with internalized BMNPs were able to magnetically concentrate in a dose-dependent manner. This behavior was previously described for a few other types of cells treated with magnetic NPs, such as N9 microglia cells, End3 endothelial cells from the brain capillary, and the DC2.4 dendritic cells [50,51,52]. We showed that, by applying an alternating magnetic field, the BMNPs heat the surrounding medium, which increases the efficiency of the drug release. These results were comparable to those observed by Lu et al. [53] in the CT-26 colon carcinoma cell line treated with magnetic graphene oxide/Fe_3_O_4_ NPs.

In the context of the interaction of BMNPs with blood cells, BMNPs did not show a significant percentage of hemolysis (<2%), being non hemolytic according to the categories of Schlenk et al. [54] (non-hemolytic (0–2%), slightly hemolytic (2–5%), or hemolytic (>5%)). In the case of WBC viability, an initial dose-dependent toxicity was observed during the first hour of BMNP exposure, although, after 12 h, the cell viability was recovered. Such a recovery after longer treatment remains unclear. At shorter time intervals, the toxicity of the BMNPs could be related to the increase of the available iron caused by the degradation of the nanoparticles. In fact, previous studies showed that the administration of iron nanoparticles can modify the immune function. For instance, since blood cells are able to synthesize iron-bearing molecules, such as transferrin, which is essential for the development of activated T lymphocytes [55], it was shown that the treatment with magnetite nanoparticles produces significant changes in the proportions of T lymphocytes in a dose-dependent manner [56]. Other authors even found differences in the proportion of T lymphocytes in peripheral blood of mice treated with low doses of magnetite nanoparticles compared to that of untreated mice [57].

Finally, the interaction between the BMNPs and macrophage cells induced a reduction in RAW 264.7 cell viability at 1 and 12 h upon treatment. Previous studies [58] showed that magnetic iron-oxide NPs cause mitochondrial damage in RAW 264.7 cells via the production of high levels of oxidative stress and the up regulation of endoplasmic reticulum (ER) stress-related genes. In our case, it could be that the massive cellular internalization and the aggregation of BMNPs by the RAW 264.7 cell line during the first hour of treatment caused great cellular damage, inducing cell death and/or inhibition of cellular replication. It was previously pointed out that the colloidal stability of magnetic nanoparticles is an important factor to consider, since their aggregation may potentially pose a risk by altering the hemostatic balance of the blood components or by causing thrombotic disorders [59].

Nevertheless, our results show that the Oxa–BMNP nanoassemblies are able to locally deliver Oxa based on changes in the environmental pH values, and to increase the toxicity of this drug by facilitating the nanoassembly’s internalization in the cells. Therefore, it is worth doing further research to reduce the agglomeration of the NPs, thus preventing thrombotic disorders and macrophage damage. Potential future lines of research may point toward the coating of NPs with polymers that increase colloidal stability, the coating of the nanoassembly with liposomes, and/or exploring alternative administration routes.

## 5. Conclusions

The results from the present study demonstrate that biomimetic magnetic nanoparticles (BMNPs) can be efficiently coupled to Oxa to form Oxa–BMNP nanoassemblies. Such a coupling is the result of the electrostatic interaction between Oxa and the surface of the NP, also favored by the cooperativity effect between the Oxa molecules. The drug loading capacity (Q_max_) is 0.41 ± 0.08 mg Oxa/mg magnetite. These nanoassemblies are stable at physiological pH values (D_R_ < 2%), but Oxa is efficiently released from the BMNPs at acidic pH values (D_R_ ~ 20%, 1 h), noticeably increased also under hyperthermia conditions (D_R_ ~ 80%, 2.5 h). While BMNPs showed good cytocompatibility in most cell lines, the Oxa–BMNP nanoassemblies increased the cytotoxic effect of the drug. Such an effect is the result of the BMNP internalization in the colon cancer cells, probably via endocytosis. Moreover, the BMNPs, once internalized, keep efficiently responding to an external continuous magnetic field, allowing the possibility of concentrating the nanoassemblies at the target site, avoiding further dispersion. BMNPs are non-hemolytic and non-toxic for WBCs, although they reduce the viability of macrophages. Further research needs to be done in order to improve the interaction between BMNPs and macrophages with the goal of potential in vivo use of the Oxa–BMNP nanoassemblies.

## Figures and Tables

**Table 1:** IC_50_ value of free oxaliplatin (Oxa) and Oxa–biomimetic magnetic nanoparticles (BMNPs) in the cell lines tested.

CELL LINE	IC_50_ FREE OXA	IC_50_ OXA–BMNPS	IC_50_ FOLD CHANGE (DECREASE)
**CCD-18**	0.66 ± 0.06	0.40 ± 0.02	1.63
**HCT-15**	1.91 ± 0.15	1.03 ± 0.05	1.85
**HT-29**	4.15 ± 0.1	1.49 ± 0.14	2.79
**MC-38**	0.49 ± 0.09	0.32 ± 0.02	1.50
**T-84**	4.30 ± 0.15	1.92 ± 0.4	2.24
**SW480**	1.68 ± 0.01	0.67 ± 0.05	2.52

Data represent the mean values ± SD of triplicate cultures.

## Supplementary Materials

The following are available online at https://www.mdpi.com/1999-4923/11/8/395/s1: Figure S1: Comparative magnetic migration assay of T-84 (A), SW840 (B), and HT29 (C) colon cancer cells exposed to different concentrations of BMNPs and Fe and stained with SRB. Migration of the cells after treatments was evaluated in the presence or absence of a magnet.

## References

[1] Bray F., Ferlay J., Soerjomataram I., Siegel R.L., Torre L.A., Jemal A. (2018). Global cancer statistics 2018: GLOBOCAN estimates of incidence and mortality worldwide for 36 cancers in 185 countries. CA Cancer J. Clin..

[2] Banerjee A., Pathak S., Subramanium V.D., Dharanivasan G., Murugesan R., Verma R.S. (2017). Strategies for targeted drug delivery in treatment of colon cancer: Current trends and future perspectives. Drug Discov. Today.

[3] Simon K. (2016). Colorectal cancer development and advances in screening. Clin. Interv. Aging.

[4] Ferlay J., Colombet M., Soerjomataram I., Dyba T., Randi G., Bettio M., Gavin A., Visser O., Bray F. (2018). Cancer incidence and mortality patterns in Europe: Estimates for 40 countries and 25 major cancers in 2018. Eur. J. Cancer.

[5] Siegel R.L., Miller K.D., Jemal A. (2018). Cancer statistics, 2018: Cancer Statistics, 2018. CA A Cancer J. Clin..

[6] Hubbard J.M., Grothey A. (2013). Adolescent and young adult colorectal cancer. J. Natl. Compr. Cancer Netw..

[7] Dienstmann R., Salazar R., Tabernero J. (2015). Personalizing colon cancer adjuvant therapy: Selecting optimal treatments for individual patients. J. Clin. Oncol..

[8] Ychou M., Rivoire M., Thezenas S., Quenet F., Delpero J.-R., Rebischung C., Letoublon C., Guimbaud R., Francois E., Ducreux M. (2013). A randomized phase II trial of three intensified chemotherapy regimens in first-line treatment of colorectal cancer patients with initially unresectable or not optimally resectable liver metastases. The METHEP trial. Ann. Surg. Oncol..

[9] Betsiou M., Sikalidis C., Papageorgiou A. (2007). Adsorption of oxaliplatin by hydroxyapatite. Bioautomation.

[10] Carrato A., Gallego J., Díaz-Rubio E. (2002). Oxaliplatin: Results in colorectal carcinoma. Crit. Rev. Oncol. Hematol..

[11] Betsiou M., Bantsis G., Zoi I., Sikalidis C. (2012). Adsorption and release of gemcitabine hydrochloride and oxaliplatin by hydroxyapatite. Ceram. Int..

[12] Dunn T.A., Schmoll H.J., Grünwald V., Bokemeyer V., Casper J. (1997). Comparative cytotoxicity of oxaliplatin and cisplatin in non-seminomatous germ cell cancer cell lines. Investig. New Drugs.

[13] Ades S. (2009). Adjuvant chemotherapy for colon cancer in the elderly: Moving from evidence to practice. Oncology.

[14] Kotelevets L., Chastre E., Desmaële D., Couvreur P. (2016). Nanotechnologies for the treatment of colon cancer: From old drugs to new hope. Int. J. Pharm..

[15] Wang A.Z., Langer R., Farokhzad O.C. (2012). Nanoparticle delivery of cancer drugs. Annu. Rev. Med..

[16] Sau S., Alsaab H.O., Bhise K., Alzhrani R., Nabil G., Iyer A.K. (2018). Multifunctional nanoparticles for cancer immunotherapy: A groundbreaking approach for reprogramming malfunctioned tumor environment. J. Control. Release.

[17] Dobson J. (2006). Magnetic micro- and nano-particle-based targeting for drug and gene delivery. Nanomedicine.

[18] Datta N.R., Krishnan S., Speiser D.E., Neufeld E., Kuster N., Bodis S., Hofmann H. (2016). Magnetic nanoparticle-induced hyperthermia with appropriate payloads: Paul Ehrlich’s “magic (nano)bullet” for cancer theranostics?. Cancer Treat. Rev..

[19] Shubayev V.I., Pisanic T.R., Jin S. (2009). Magnetic nanoparticles for theragnostics. ‎Adv. Drug Deliv. Rev..

[20] Pankhurst Q.A., Connolly J., Jones S.K., Dobson J. (2003). Applications of magnetic nanoparticles in biomedicine. J. Phys. D Appl. Phys..

[21] Prozorov T., Bazylinski D.A., Mallapragada S.K., Prozorov R. (2013). Novel magnetic nanomaterials inspired by magnetotactic bacteria: Topical review. Mater. Sci. Eng. R Rep..

[22] Karimi M., Ghasemi A., Sahandi Zangabad P., Rahighi R., Moosavi Basri S.M., Mirshekari H., Amiri M., Shafaei Pishabad Z., Aslani A., Bozorgomid M. (2016). Smart micro/nanoparticles in stimulus-responsive drug/gene delivery systems. Chem. Soc. Rev..

[23] Alphandéry E., Faure S., Raison L., Duguet E., Howse P.A., Bazylinski D.A. (2011). Heat production by bacterial magnetosomes exposed to an oscillating magnetic field. J. Phys. Chem. C.

[24] Kolhatkar A.G., Jamison A.C., Litvinov D., Willson R.C., Lee T.R. (2013). Tuning the magnetic properties of nanoparticles. Int. J. Mol. Sci..

[25] Amemiya Y., Arakaki A., Staniland S.S., Tanaka T., Matsunaga T. (2007). Controlled formation of magnetite crystal by partial oxidation of ferrous hydroxide in the presence of recombinant magnetotactic bacterial protein Mms6. Biomaterials.

[26] Prozorov T., Mallapragada S.K., Narasimhan B., Wang L., Palo P., Nilsen-Hamilton M., Williams T.J., Bazylinski D.A., Prozorov R., Canfield P.C. (2007). Protein-mediated synthesis of uniform superparamagnetic magnetite nanocrystals. Adv. Funct. Mater..

[27] Staniland S.S., Rawlings A.E. (2016). Crystallizing the function of the magnetosome membrane mineralization protein Mms6. Biochem. Soc. Trans..

[28] Valverde-Tercedor C., Montalbán-López M., Perez-Gonzalez T., Sanchez-Quesada M.S., Prozorov T., Pineda-Molina E., Fernandez-Vivas M.A., Rodriguez-Navarro A.B., Trubitsyn D., Bazylinski D.A. (2015). Size control of in vitro synthesized magnetite crystals by the MamC protein of *Magnetococcus marinus* strain MC-1. Appl. Microbiol. Biotechnol..

[29] Nudelman H., Valverde-tercedor C., Kolusheva S., Perez T., Widdrat M., Grimberg N., Levi H., Nelkenbaum O., Davidov G., Faivre D. (2016). Structure—Function studies of the magnetite-biomineralizing magnetosome-associated protein MamC. J. Struct. Biol..

[30] Lopez-Moreno R., Fernández-Vivas A., Valverde-Tercedor C., Azuaga Fortes A.I., Casares Atienza S., Rodriguez-Navarro A.B., Zarivach R., Jimenez-Lopez C. (2017). Magnetite nanoparticles biomineralization in the presence of the magnetosome membrane protein MamC: Effect of protein aggregation and protein structure on magnetite formation. Cryst. Growth Des..

[31] García Rubia G., Peigneux A., Jabalera Y., Puerma J., Oltolina F., Elert K., Colangelo D., Gómez Morales J., Prat M., Jimenez-Lopez C. (2018). pH-Dependent adsorption release of doxorubicin on MamC-biomimetic magnetite nanoparticles. Langmuir.

[32] Dutta R.K., Sahu S. (2012). Development of oxaliplatin encapsulated in magnetic nanocarriers of pectin as a potential targeted drug delivery for cancer therapy. Results Pharma Sci..

[33] Munaweera I., Shi Y., Koneru B., Saez R., Aliev A., Di Pasqua A.J., Balkus K.J. (2015). Chemoradiotherapeutic Magnetic Nanoparticles for Targeted Treatment of Nonsmall Cell Lung Cancer. Mol. Pharm..

[34] Martín J.D. (2004). XPowder, a Software Package for Powder X-ray Diffraction Analysis. Legal Deposit GR 1001/04. http://www.xpowder.com.

[35] Ahmed K., Tabuchi Y., Kondo T. (2015). Hyperthermia: An effective strategy to induce apoptosis in cancer cells. Apoptosis.

[36] Ortiz R., Cabeza L., Arias J.L., Melguizo C., Álvarez P.J., Vélez C., Clares B., Áranega A., Prados J. (2015). Poly(butylcyanoacrylate) and Poly(ε-caprolactone) Nanoparticles Loaded with 5-Fluorouracil Increase the Cytotoxic Effect of the Drug in Experimental Colon Cancer. AAPS J..

[37] Evans B.C., Nelson C.E., Yu S.S., Beavers K.R., Kim A.J., Li H., Nelson H.M., Giorgio T.D., Duvall C.L. (2013). Ex vivo red blood cell hemolysis assay for the evaluation of pH-responsive endosomolytic agents for cytosolic delivery of biomacromolecular drugs. J. Vis. Exp..

[38] Assadian E., Zarei M.H., Gilani A.G., Farshin M., Degampanah H., Pourahmad J. (2017). Toxicity of copper oxide (CuO) nanoparticles on human blood lymphocytes. Biol. Trace Elem. Res..

[39] Lorente C., Cabeza L., Clares B., Ortiz R., Halbaut L., Delgado Á.V., Perazzoli G., Prados J., Arias J.L., Melguizo C. (2018). Formulation and in vitro evaluation of magnetoliposomes as a potential nanotool in colorectal cancer therapy. Colloids Surf. B Biointerfaces.

[40] Iafisco M., Drouet C., Adamiano A., Pascaud P., Montesi M., Panseri S., Sarda S., Tampieri A. (2016). Superparamagnetic iron-doped nanocrystalline apatite as a delivery system for doxorubicin. ‎J. Mater. Chem. B.

[41] Wu S., Zhao X., Li Y., Du Q., Sun J., Wang Y., Wang X., Xia Y., Wang Z., Xia L. (2013). Adsorption properties of doxorubicin hydrochloride onto graphene oxide: Equilibrium, kinetic and thermodynamic studies. Materials.

[42] Geisow M.J., Evans W.H. (1984). pH in the endosome: Measurements during pinocytosis and receptor-mediated endocytosis. ‎Exp. Cell Res..

[43] Iglesias G.R., Reyes-Ortega F., Checa Fernandez B.L., Delgado Á.V. (2018). Hyperthermia-Triggered Gemcitabine Release from Polymer-Coated Magnetite Nanoparticles. Polymers.

[44] Felfoul O., Mohammadi M., Taherkhani S., de Lanauze D., Zhong Xu Y., Loghin D., Essa S., Jancik S., Houle D., Lafleur M. (2016). Magneto-aerotactic bacteria deliver drug-containing nanoliposomes to tumour hypoxic regions. Nat. Nanotechnol..

[45] Taherkhani S., Mohammadi M., Daoud J., Martel S., Tabrizian M. (2014). Covalent Binding of Nanoliposomes to the Surface of Magnetotactic Bacteria for the Synthesis of Self-Propelled Therapeutic Agents. ACS Nano.

[46] Manna P.T., Obado S.O., Boehm C., Gadelha C., Sali A., Chait B.T., Rout M.P., Field M.C. (2017). Lineage-specific proteins essential for endocytosis in trypanosomes. J. Cell Sci..

[47] Mercanti V., Marchetti A., Lelong E., Perez F., Orci L., Cosson P. (2010). Transmembrane domains control exclusion of membrane proteins from clathrin-coated pits. J. Cell Sci..

[48] Bae J.-E., Huh M.-I., Ryu B.-K., Do J.-Y., Jin S.-U., Moon M.-J., Jung J.-C., Chang Y., Kim E., Chi S.-G. (2011). The effect of static magnetic fields on the aggregation and cytotoxicity of magnetic nanoparticles. Biomaterials.

[49] Nosrati H., Salehiabar M., Manjili H.K., Danafar H., Davaran S. (2018). Preparation of magnetic albumin nanoparticles via a simple and one-pot desolvation and co-precipitation method for medical and pharmaceutical applications. Int. J. Biol. Macromol..

[50] White E.E., Pai A., Weng Y., Suresh A.K., Van Haute D., Pailevanian T., Alizadeh D., Hajimiri A., Badie B., Berlin J.M. (2015). Functionalized iron oxide nanoparticles for controlling the movement of immune cells. Nanoscale.

[51] Philosof-Mazor L., Dakwar G.R., Popov M., Kolusheva S., Shames A., Linder C., Greenberg S., Heldman E., Stepensky D., Jelinek R. (2013). Bolaamphiphilic vesicles encapsulating iron oxide nanoparticles: New vehicles for magnetically targeted drug delivery. Int. J. Pharm..

[52] Jin H., Qian Y., Dai Y., Qiao S., Huang C., Lu L., Luo Q., Chen J., Zhang Z. (2016). Magnetic enrichment of dendritic cell vaccine in lymph node with fluorescent-magnetic nanoparticles enhanced cancer immunotherapy. Theranostics.

[53] Lu Y.-J., Lin P.-Y., Huang P.-H., Kuo C.-Y., Shalumon K.T., Chen M.-Y., Chen J.-P. (2018). Magnetic graphene oxide for dual targeted delivery of doxorubicin and photothermal therapy. Nanomaterials.

[54] Schlenk F., Werner S., Rabel M., Jacobs F., Bergemann C., Clement J.H., Fischer D. (2017). Comprehensive analysis of the in vitro and ex ovo hemocompatibility of surface engineered iron oxide nanoparticles for biomedical applications. Arch. Toxicol..

[55] Lum J.B., Infante A.J., Makker D.M., Yang F., Bowman B.H. (1986). Transferrin synthesis by inducer T lymphocytes. J. Clin. Investig..

[56] Wang J., Chen B., Jin N., Xia G., Chen Y., Zhou Y., Cai X., Ding J., Li X., Wang X. (2011). The changes of T lymphocytes and cytokines in ICR mice fed with Fe_3_O_4_ magnetic nanoparticles. Int. J. Nanomed..

[57] Chen B.-A., Jin N., Wang J., Ding J., Gao C., Cheng J., Xia G., Gao F., Zhou Y., Chen Y. (2010). The effect of magnetic nanoparticles of Fe_3_O_4_ on immune function in normal ICR mice. Int. J. Nanomed..

[58] Park E.-J., Choi D.-H., Kim Y., Lee E.-W., Song J., Cho M.-H., Kim J.-H., Kim S.-W. (2014). Magnetic iron oxide nanoparticles induce autophagy preceding apoptosis through mitochondrial damage and ER stress in RAW264.7 cells. ‎Toxicol. In Vitro.

[59] Ilinskaya A.N., Dobrovolskaia M.A. (2013). Nanoparticles and the blood coagulation system. Part II: Safety concerns. Nanomedicine.

