# Supplementary material for: Oxaliplatin–Biomimetic Magnetic Nanoparticle Assemblies for Colon Cancer-Targeted Chemotherapy: An In Vitro Study
Source: Pharmaceutics. 2019 Aug 6;11(8):395. doi: 10.3390/pharmaceutics11080395 (PMC6723246; doi:10.3390/pharmaceutics11080395)
Supplement: Supplementary file 1 [file pharmaceutics-11-00395-s001.pdf]

SupplementaryMaterials: Oxaliplatin-biomimetic magnetic nanoparticles assemblies for colon cancer-targeted chemotherapy: an *in vitro* study

Ylenia Jabalera, Beatriz Garcia-Pinel ,Raul Ortiz , Guillermo Iglesias, Laura Cabeza , José Prados, Concepción Jimenez-Lopez and Consolación Melguizo

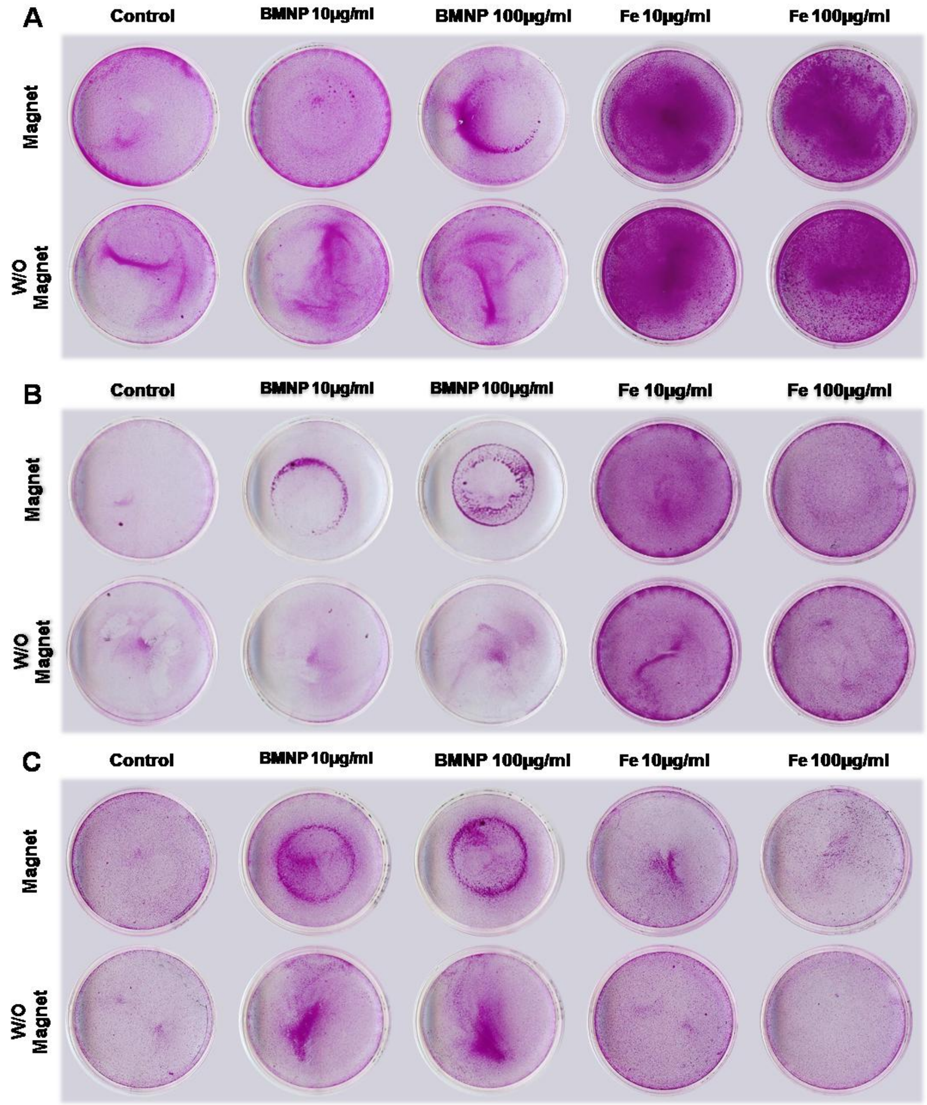

Figure S1. Comparative magnetic migration assay of T-84 (A), SW840 (B) and HT29 (C) colon cancer cells exposed to different concentrations of BMNPs and Fe and stained with SRB. Migration of the cells after treatments was evaluated in the presence or absence of a magnet.
